# Supplementary material for: Ultrafast spin exchange-coupling torque via photo-excited charge-transfer processes
Source: Nat Commun. 2015 Oct 28;6:8800. doi: 10.1038/ncomms9800 (PMC4640140; doi:10.1038/ncomms9800)
Supplement: Supplementary Information — Supplementary Figures 1-4, Supplementary Table 1, Supplementary Notes 1-5, and Supplementary References. [file ncomms9800-s1.pdf]

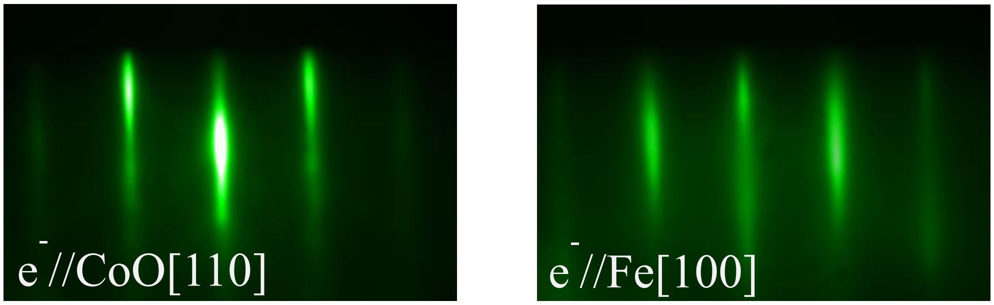

Supplementary Figure 1 | Structure characterization. Reflection high-energy electron diffraction (RHEED Patterns of CoO / MgO(001) and Fe / CoO / MgO (001).

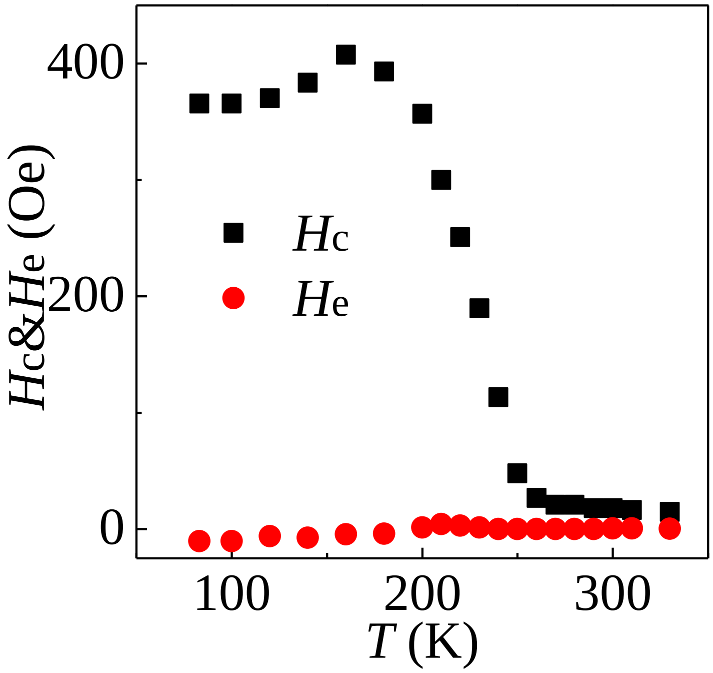

Supplementary Figure 2 | Static magnetic properties. Temperature dependence of coercivity (H_c) and exchange biasing field (H_e) obtained from easy axis hysteresis loops with magneto-optical Kerr effect (MOKE).

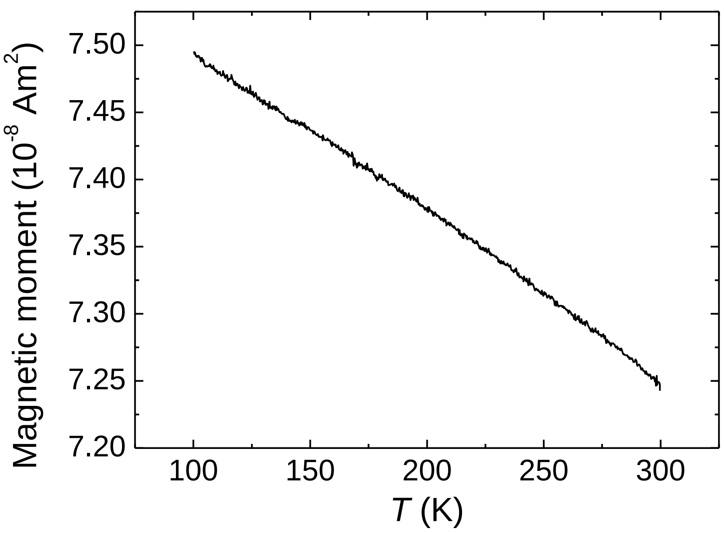

Supplementary Figure 3 | Magnetic moment. Temperature dependence of total magnetic moment measured with superconducting quantum interference device SQUID magnetometry.

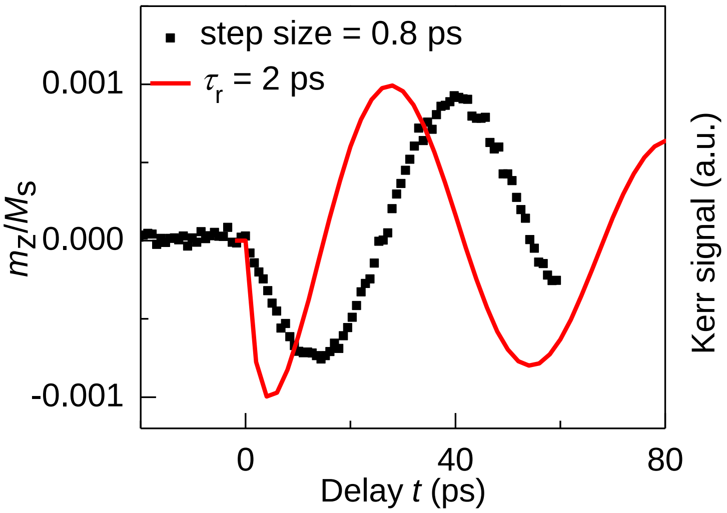

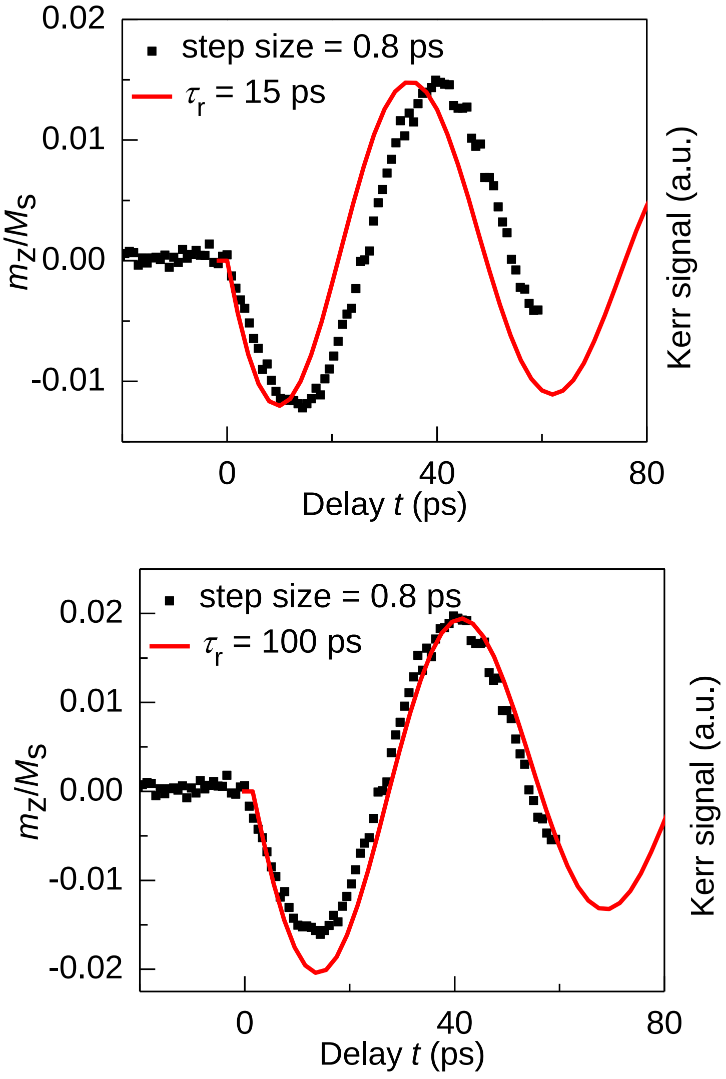

Supplementary Figure 4 | Real-time simulation results of magnetization component change. Measured Kerr signal change (black squares), simulated polar magnetization component (red curve) as a function of time delay t with recovery time 2 ps (**a**), 15 ps (**b**) and 100 ps (**c**).

Supplementary Table 1 | Magnetic anisotropies at different temperatures.

T (K)	H_d^{eff} (T)	K_u/M_S (Oe)	K_1/M_S (Oe)
300	2.00±0.02	205±3	290±3
240	2.01±0.02	942±9	295±3
100	2.08±0.02	2050±20	314±3

Supplementary Note 1 | Sample characterization

The high-quality epitaxial growth of the CoO and Fe films is revealed by the reflection high-energy electron diffraction (RHEED) patterns, as shown in Supplementary Figure 1. The epitaxial relation is CoO[110]//Fe[100]. The samples are covered with a 3-nm thick MgO protection layer before being taken out of the growth chamber.

The samples are characterized by the magneto-optical Kerr effect (MOKE). Longitudinal MOKE measurements are carried out with external magnetic field applied along the Fe [100] and [010] directions. The results indicate a negligible exchange bias (H_e) in Fe/CoO from 80 K to above RT, as shown in Supplementary Figure 2 and Fig. 2(a) in the main text. The absence of exchange bias may be due to insufficient uncompensated AFM spins at the interface in the epitaxial sample with the nearly ideal structure, as the exchange-bias effect in Fe/CoO should be sensitive to the stoichiometry and the degradation of a compensated surface [Supplementary Reference 1]. Moreover, the blocking temperature of a 10-nm thick CoO layer is found to be 110 K [Supplementary Reference 2]. Thus for the 3-nm thick CoO films discussed in this study, a significantly lower blocking temperature can be expected. Nevertheless, the field cooling leads to the alignment of AFM spins perpendicular to the FM Fe spins as a result of spin flop coupling. Due to the exchange coupling of the Fe magnetization with the AFM spins, a uniaxial anisotropy K_u appears, and the coercivity (H_c) increases with decreasing temperature down to 160 K, below which H_c decreases because less AFM spins are dragged to switch with FM spins. As shown in Fig. 2(a) in the main text, the hysteresis loops show perfect squareness for the field along the easy axis; however, it is hard to reach saturation magnetization at the largest field, restricted by our electromagnet, along the hard axis perpendicular to the cooling field. These results indicate a well-defined easy axis and homogeneous anisotropy of the sample.

Supplementary Figure 3 shows that the total magnetic moment of Fe layer in Fe/CoO thin film structure remains almost unchanged from 100 to 300 K, which is measured with a superconducting quantum interference device (SQUID). The decrease of M_S at higher T is within 4%, indicating that the Curie temperature of the Fe thin film layer is much higher than 300 K.

Supplementary Note 2 | Magnetic anisotropies

We determine the magnetic anisotropies with Supplementary Equation 1. Along the equilibrium direction, F reaches the minimum in Supplementary Equation 2. Therefore, θ turns out to be 90° and ϕ can be expressed as a function of anisotropy fields and H. The dependence of f on H can be well fitted with the dispersion relationship in Supplementary Equation 1, and the values of K_u and K_1 are derived.

Supplementary Table 1 summarizes the values of magnetic anisotropies at certain temperatures T. $K_{\perp} > 0$ denotes the perpendicular magnetic anisotropy from Fe/oxides interfaces as recently discovered [Supplementary Reference 3]. It is derived in Supplementary Reference 4 that $H_d^{eff} = H_d - 2K_{\perp}/M_S = 2.00 \pm 0.02$ T for the same Fe/CoO thin film at room temperature, indicating that $K_{\perp}/M_s$ is about 0.5 kOe. Therefore, the derived K_s at Fe/oxide interface is around 0.17 mJ/m^2, which is consistent with the calculated value for over-oxidized interface [Supplementary Reference 5] and other experiments [Supplementary Reference 3]. When T decreases to 78K, H_d^{eff} increases slightly to 2.08 ± 0.02 T, due to the increase of M_S or modulation of $K_{\perp}$.

Although the Fe 3d/O 2p hybridization at the interface may result in the perpendicular magnetic anisotropy $K_{\perp}$, the Fe-O bond formation at the Fe/CoO interface is not responsible for the enhanced uniaxial magnetic anisotropy K_u because we found that K_u of the Fe film grown on 1-nm-thick CoO is very similar to that of the Fe film directly grown on MgO. The CoO thickness must be large enough to establish the AFM order and form exchange coupling with the Fe spins. Since the spin precession excitation is correlated with the modulations of the CoO AFM spins and the resultant exchange coupling-induced anisotropy, we may exclude the role of Fe-O bond in the excitation mechanism. Moreover, we did not observe apparent temperature dependence of the spin precession amplitude in the Fe/MgO (001) capped with a 3-nm-thick MgO layer, so we believe that the modulation of perpendicular magnetic anisotropy due to the MgO layer does not play an important role in the spin precession excitation. We also note that the MgO has the optical band gap of ~7.8 eV and its absorption of 400-nm light is extremely small, so the 3-nm-thick capping layer is nearly transparent for 400 nm. We thus may neglect the contribution of 400-nm light absorption by the MgO capping layer to the spin precession excitation.

Supplementary Note 3 | Simulation of precession amplitude

We simulate the dependence of spin precession amplitude A on H and T. ϕ can be expressed as a function of anisotropy fields and H, through minimizing the magnetic free energy in Supplementary Equation 2. If the duration of K_u change is comparable to the magnetization precession period, A can be assumed to be proportional to the equilibrium direction change by modulation of K_u (ΔK_u) [Supplementary Reference 6-8, Supplementary Equation 3]. The scaling pre-factor s represents the convention from magnetization precession amplitude to detected Kerr signal. First, the H dependence of A is fitted with parameters ΔK_u and s. Then for the temperature dependence of A, ΔK_u is parameterized as $K_u(T)$-$K_u(T$+20K), and s is set as derived from the H dependence simulation.

Since we do not have the K_u values above 300K, we extend the values of K_u to higher temperature from the fitted dependence of K_u on T in Fig. 3(c) of main text. The simulation on temperature dependence of A above 280K is based on those values. The presented A at 300 K is derived from fitting the field dependence of A shown in Fig. 3(b) of main text.

Supplementary Note 4 | Estimation of thermal effect

The conventional heat diffusion from Fe layer to CoO layer can not explain the fast excitation mechanism, because the heat diffusion is too slow and the 400-nm pump-induced heat of Fe layer ($\Delta T \approx 15K$) will mainly diffuse to the MgO layer, since the heat conduction is much higher in the Fe and MgO layers than in the CoO layer.

The direct heating of CoO layer by the blue light is also negligible, i.e. $\Delta T \approx 1K$. The temperature raise in CoO layer ΔT caused by instant laser pulse heating is estimated through $E = C\rho t\pi r^2 \Delta T_l/M$, where E is the light energy absorbed, C the molar heat capacity, ρ the density, t the thickness of layer, r the radius of pumped region and M the molar mass. In the calculation of E, the reflection from Fe surface and Fe/CoO interface and absorption in Fe layer are also considered.

Supplementary Note 5 | Simulation of recovery time

Real-time simulation of magnetization precession is carried out with LLG equations with time interval Δt=0.2ps in Supplementary Equations 4 and 5.

Supplementary Figure 4 displays the simulation results with other τ_r values, where shorter or longer τ_r leads to mismatch in the oscillation phase. Generally speaking, the longer τ_r postpone the phase of oscillation, and the precession frequency during the τ_r is smaller compared with that after K_u is recovered.

Supplementary Equation 1 | Field (H) dependence of spin precession frequency (f)

$$2\pi \cdot f = \gamma\sqrt{[H\cos(\delta-\phi)+H^{\alpha}][H\cos(\delta-\phi)+H^{\beta}]}$$

where $H^{\alpha} = H_d^{eff} - 2K_u\sin^2\phi/M_S + K_1(2-\sin^2 2\phi)/M_S$, $H^{\beta} = 2K_1\cos4\phi/M_S + 2K_u\cos2\phi/M_S$, with effective demagnetization field $H_d^{eff} = 4\pi M_S - 2K_{\perp}/M_S$, saturated magnetization M_S, and gyromagnetic ratio $\gamma = \gamma_e g/2$ (for Fe, g = 2.09 and $\gamma_e = 1.76\times10^7$ Hz/Oe). ϕ and δ are the angles of in-plane equilibrium magnetization and H with respect to the Fe axis [100].

K_u, K_1 and $K_\perp$ are the in-plane uniaxial, crystalline cubic, and out-of-plane magnetic anisotropies, respectively.

Supplementary Equation 2 | Magnetic free energy (*F*)

$$F = \frac{K_1}{4}(\sin^4\theta\sin^2 2\phi + \sin^2 2\theta) + K_u \sin^2\phi\sin^2\theta + K_\perp\cos^2\theta + 2\pi(M\cos\theta)^2$$
$$- HM(\sin\theta\cos\phi + \sin\theta\sin\phi)$$

where the θ is the angle between the equilibrium direction of magnetization and Fe [001] direction.

Supplementary Equation 3 | Simulation of spin precession amplitude (*A*)

$$A = s(\phi(K_u) - \phi(K_u - \varDelta K_u)).$$

The scaling pre-factor *s* represents the convention from magnetization precession amplitude to detected Kerr signal.

Supplementary Equations 4 and 5 | Real time simulation of spin precession

$$m_z(t+\Delta t)/M_S = m_z(t)/M_S \times \exp(1-\Delta t/\tau) - m_y(t)/M_S \times \gamma\,(H\cos(\delta-\phi) + H^{\beta}(t))\times\Delta t$$

$$m_y(t+\Delta t)/M_S = m_y(t)/M_S \times \exp(1-\Delta t/\tau) + m_z(t+\Delta t)\times\gamma\,(H\cos(\delta-\phi) + H^{\alpha}(t))\times\Delta t + (\phi(K_u - \Delta K_u(t+\Delta t)) - \phi(K_u - \Delta K_u(t)))$$

where the first term represents the Gilbert damping effect and the second term denotes the circling of spin precession. As for m_y, there is an additional term describing the change of equilibrium direction driven by modulation of K_u.

Supplementary References:

[1] N. J. Gökemeijer, R. L. Penn, D. R. Veblen, and C. L. Chien, Exchange coupling in epitaxial CoO/NiFe bilayers with compensated and uncompensated interfacial spin structures. Phys. Rev. B.63.174422 (2001)

[2] J. Miguel, R. Abrudan, M. Bernien, M. Piantek, C. Tieg, J. Kirschner and W. Kuch, Effect of Exchange Bias on Magnetic Anisotropies in Fe/CoO Bilayers. J. Supercond. Nov. Magn. (2012) 25:2597-2603

[3] T. Maruyama, Y. Shiota, T. Nozaki, K. Ohta, N. Toda, M. Mizuguchi, AA. Tulapurkar, T. Shinjo, M. Shiraishi, S. Mizukami, Y. Ando and Y. Suzuki, Large voltage-induced magnetic anisotropy change in a few atomic layers of iron. Nat. Nano. 4, 158-161 (2009)

[4] Fan Y, et al. Photoinduced spin angular momentum transfer into an antiferromagnetic insulator. Physical Review B 89, 094428 (2014).

[5] A. Hallal, HX. Yang, B. Dieny, and M. Chshiev, Anatomy of perpendicular magnetic anisotropy in Fe/MgO magnetic tunnel junctions: First-principles insight. Phys. Rev. B 88, 184423 (2013)

[6] Liu Y, et al. Optically induced magnetization dynamics and variation of damping parameter in epitaxial Co2MnSi Heusler alloy films, Phys. Rev. B 81, 094402 (2010).

[7] Hansteen F, Kimel A, Kirilyuk A, Rasing T. Femtosecond Photomagnetic Switching of Spins in Ferrimagnetic Garnet Films. Physical Review Letters 95, 047402 (2005).

[8] Kirilyuk A, Kimel AV, Rasing T. Ultrafast optical manipulation of magnetic order. Reviews of Modern Physics 82, 2731-2784 (2010).
